# Supplementary figures and images for: Not just trash birds: Quantifying avian diversity at landfills using community science data
Source: PLoS One. 2021 Sep 27;16(9):e0255391. doi: 10.1371/journal.pone.0255391 (PMC8476020; doi:10.1371/journal.pone.0255391)

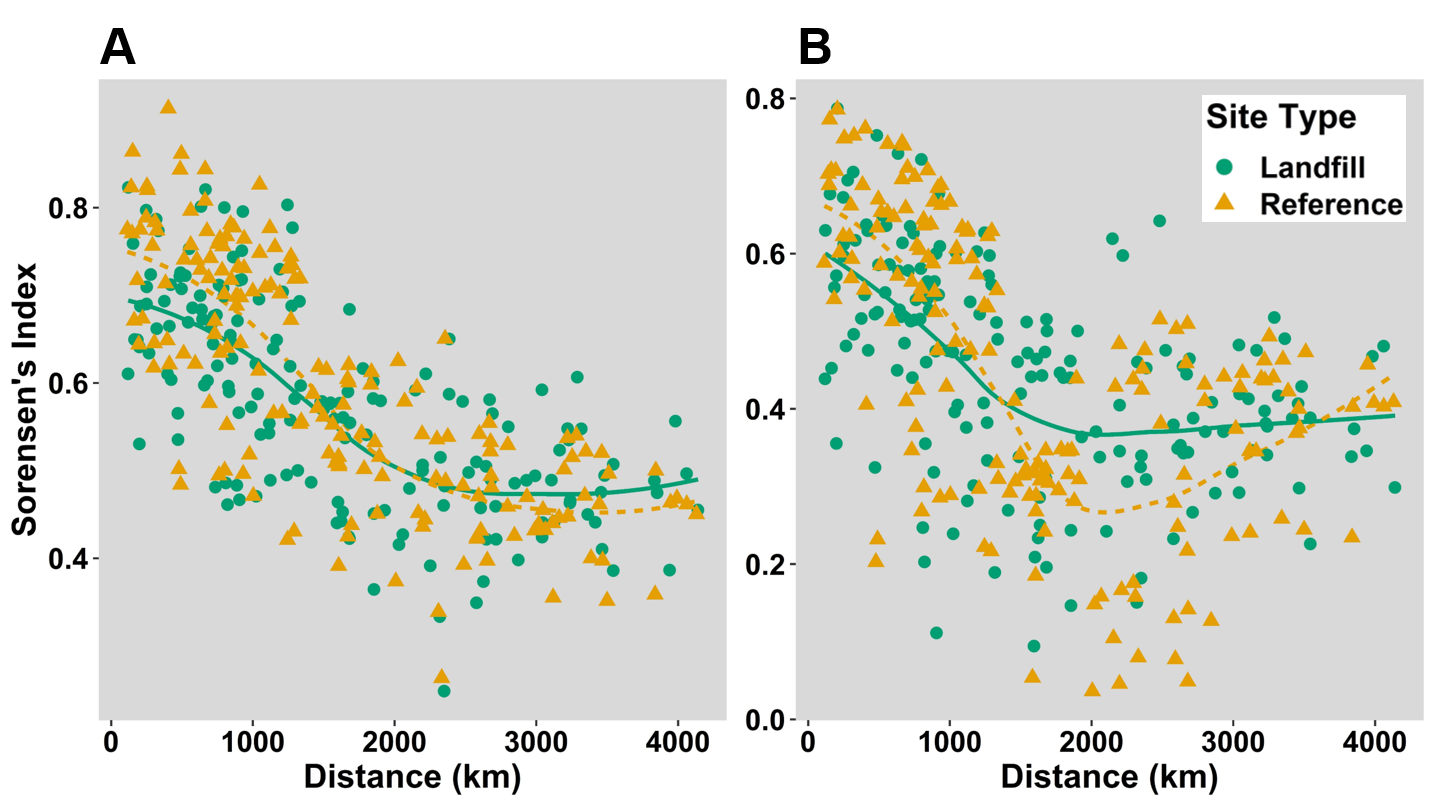

Supplement: S1 Fig — Changes in community similarity (measured by Sorensen’s Index) between pairs of landfills and pairs reference sites, plotted as a function of inter-site distances. Lines represent a spline fit. (TIF) [file pone.0255391.s001.tif]

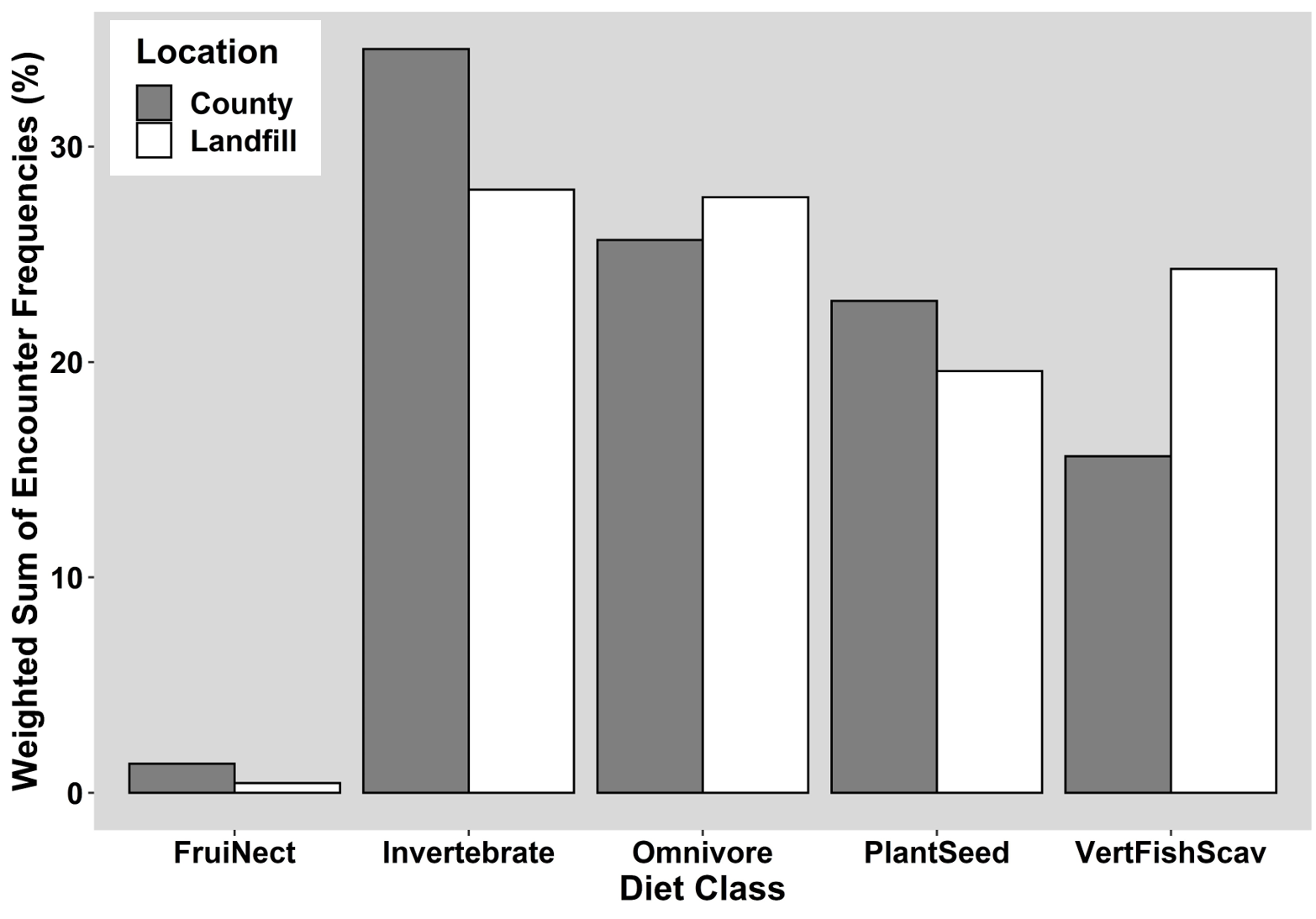

Supplement: S2 Fig — Frequency of all species’ diet classes at landfills (white) compared to background county-level species pools (grey). (TIF) [file pone.0255391.s002.tif]
