# Supplementary material for: Not just trash birds: Quantifying avian diversity at landfills using community science data
Source: PLoS One. 2021 Sep 27;16(9):e0255391. doi: 10.1371/journal.pone.0255391 (PMC8476020; doi:10.1371/journal.pone.0255391)
Supplement: S1 Table — American states are identified using two letter postal abbreviations. Checklist No. represents the total number of checklists submitted at each hotspot. Hotspot names appear exactly how they do in the eBird database. (PDF) [file pone.0255391.s003.pdf]

State	County	Checklist No.	Landfill Hotspot	Checklist No.	Reference Site Hotspot	Checklist No.
ID	Ada	51706	Ada County Landfill	454	Boise State Intermountain Bird Observatory Riverside Study Site	462
UT	Cache	26699	Logan Landfill	153	Newton Reservoir -- South End Access	150
TX	Cameron	91106	Brownsville Landfill (LTC 041)	1225	South Padre Is.--Bay Access mudflats N. of Conv Ctr.	1224
GA	Clarke	28377	Athens-Clarke Co. Landfill	359	Whitehall Forest	386
OH	Cuyahoga	85044	Solon Landfill	107	Bradley Woods Reservation	106
WI	Douglas	28514	Superior Landfill	773	Connors Point	747
IN	Elkhart	19710	Elkhart County Landfill and Pond	105	Pumpkinvine Nature Trail--South of IN4	104
OH	Hancock	11522	Hancock County Sanitary Landfill Wetland	148	Clay Pits--Strawbridge Pond	148
CT	Hartford	93206	Manchester Landfill & Laurel Marsh	221	Suffield WMA	218
WI	Jefferson	16602	Johnson Creek Landfill	378	Glacial Drumlin State Trail--Lake Mills west to Zeloski Marsh	359
NY	Kings	65959	Fountain Avenue Landfill	103	Prospect Park--Upper Pool	104
VA	Northampton	23994	Cheriton Landfill	715	Oyster	711
PA	Northampton	21275	Grand Central Landfill	267	Ballas Park	272
FL	Okaloosa	9668	Okaloosa Landfill	386	Destin--west jetty	319
NY	Onondaga	43342	DeWitt Marsh and Landfill	224	Jamesville Beach County Park	239
MI	Sanilac	3274	Tri-City Landfill (Sanilac Co.)	180	Flynn Twp. Nature Center	146
FL	Sarasota	61693	CCSWDC Landfill	288	Quick Point Nature Preserve	296
MI	Washtenaw	96435	Ann Arbor Landfill/Wheeler Service Center	787	Crooked Lake	769
CA	Yolo	54412	Yolo County Central Landfill pond--from levee road by CR28H	404	West Davis pond	405
