# Supplementary material for: Not just trash birds: Quantifying avian diversity at landfills using community science data
Source: PLoS One. 2021 Sep 27;16(9):e0255391. doi: 10.1371/journal.pone.0255391 (PMC8476020; doi:10.1371/journal.pone.0255391)
Supplement: S2 Table — A buffer around the center of each site was chosen with a four-kilometer radius. The NLCD data uses a 16-class classification system [54], which we have reclassified into seven more bird relevant classes. NLCD classes 12 (Perennial Ice/Snow), 72 (Sedge/Herbaceous), 73 (Lichens), and 74 (Moss) did not appear in any of our sites. All of the developed landcover classes (classes 21–24) were grouped into one class labeled “Developed.” The three forest classes (41–43) were aggregated into one class labeled “Forested.” Classes 51–52, 71, and 81 were all counted as “Grassland” landcover. Classes 90 and 95 were grouped into the “Wetlands” class. The remaining classes (11, 31, and 82) were not aggregated with other land cover classes. We present the proportion of each of our reclassified land cover classes for all landfills and all reference sites. (PDF) [file pone.0255391.s004.pdf]

Landcover Category	Percent Landcover (%)	
	Landfill	Reference Site
Barren	0.8	1.0
Crops	23.8	22.7
Developed	30.9	27.6
Forested	13.8	14.1
Grassland	13.7	8.9
Open Water	5.8	19.2
Wetland	11.1	6.6
