# Supplementary material for: Not just trash birds: Quantifying avian diversity at landfills using community science data
Source: PLoS One. 2021 Sep 27;16(9):e0255391. doi: 10.1371/journal.pone.0255391 (PMC8476020; doi:10.1371/journal.pone.0255391)
Supplement: Attachment — Submitted filename: Response to Reviewers.docx [file pone.0255391.s006.docx]

**Response to reviewer comments**

Reviewer comments are in italics; indented text denotes our response to each comment. All line numbers refer to the document titled “Revised Manuscript with Track Changes”

**Reviewer 1:**

*The authors used eBird data to compare bird communities between landfills and reference sites. They found that the landfills across the USA could provide benefits for some avian species with conservation concerns. This is an interesting paper and can provide implications for bird conservations at these landfills. I think the paper can be accepted while considering the following comments.*

Thank you

*Lines 39-40, provide the name of two declining species*

The names of the species were added (lines 40, 41)

*Line 133, provide details about the environmental conditions at these 19 landfills, e.g. in a supplementary table.*

We now report the percentage land cover of bird-relevant land cover types within a 4km buffer of the center of all landfill and all reference sites in a supplemental table (S2 Table). We have added a sentence to the manuscript that references this supplement (lines 171-173

*Line 134, how were the reference sites selected? What kind of environments were there? Habitats or environments in the reference sites could significantly determine the avian compositions. In addition, there are few comparisons between studies on bird use of landfills or species compositions.*

Reference sites were only selected based on geographical proximity (within the same county) and how frequently they were sampled (i.e. number of submitted checklists). To clarify this, we have added the sentence “Because eBird hotspot boundaries, and how much area of each site is accessible to observers, are not clearly defined, we did not include any criteria relating to habitat types in the selection of reference sites” (lines 167-169). We agree that the habitats represented by reference sites could significantly affect the species composition, and we note in the discussion that this is a limitation of our study (lines 389-390). However, in some of our analyses (e.g. proportion of dietary types represented) we avoid this problem by comparing landfills to the background (county-level) species pool.

**Review 2**

*Comments:*

*I think that the authors did a great job with this study. The objective is clear, the study was well design and the hypothesis to be tested are explicit. The authors made a very good coverage of relevant literature about the subject and make explicit and clear the research problem as well the relevance of the study for understanding how birds are responding to novel human land uses, in this case landfills and I found broadly relevant for the fields of urban ecology and land planning in urban areas. The manuscript is very well written, and the phrasing is clear. Paragraphs are well structured and logically connected. Methodology sections is fairly complete, and results are presented in a concise way. Discussion is well focused and developed. Overall, I consider the manuscript of great value. I congrats the authors for the hard effort invested in the preparation of the study. Nevertheless, I have a series of concerns about the manuscript, particularly the analytical part (statistical analysis) that I consider should be approached to make the study more robust and easily replicable in future studies. Also below I point out to particular aspects of the manuscript per section.*

We appreciate the detailed comments from this reviewer, and we have attempted to address all of the major concerns in our revised manuscript.

*TITLE:*

*I would skip: Not just trash birds. It sounds a little bit necessary this part in the title use trash birds.*

We included “trash birds” in the title as it is a term used by birdwatchers to describe common, human-associated bird species. We believe that having a distinctive title draws attention to the work. Reviewer 3 also raised this point, saying the paper has “an interesting title with high potential to be used in scientific communication”. As a result, it is our preference to keep the title as is.

*INTRODUCTION:*

*General comments:*

*1.I think that the instead refers to wildlife in the introduction, author can use the term “birds”, because this paper is devoted to birds.*

We have changed the term “wildlife” to “bird” (lines 26, 29, 63, 92) “birds” (lines 37, 50, 61, 316) or “species” (line 28) depending on context.

*2.I believe that the transition to the research problem in the third paragraph is a little bit abrupt. I consider that although it provides the arguments concerned to habitat for threatened species and benefits for some particular bird clades (gulls), the justification for the work can include more applied arguments and not only their perceived benefits to some avian groups, what are the applications for management, restoration etc. of knowing the diversity in landfills?*

We have rewritten this paragraph (lines 78-96) to highlight applied arguments (e.g. that standard landfill management in the US requires that landfill caps must be maintained as grasslands for many years), and emphasize constraints on post-closure uses (e.g. parks, hiking trails, and nature preserves). Finally, we state that a more holistic understanding of bird communities, not just species using active trash disposal sites, is a necessary first step for biodiversity-friendly management both before and after landfill closure (lines 93-96)

*3.I would include in the introduction more studies which have compared the diversity of landfill and other land use types and not only include studies showing the positive and negative effects of landfills on bird diversity or particular species. It is important to make this, because it gives context in the introduction about the necessity to make these comparisons.*

We have included more discussion on previous studies on landfill bird communities (lines 64-67)

*4. Another important point for the introduction is that important aspects of the predictions of the hypothesis are not completely developed in the introduction. Therefore, is not clear to the reader why should we expect lower turnover in pairwise comparisons of landfill sites compared to reference sites?.  Why should we expect more generalist species? Or omnivorous species? Etc. I think that literature (which is extensive for urban bird communities) can be used in the introduction to highlight these points and guide the reader to the logic or the raison d'être of the hypothesis of this study.*

We have more clearly developed the introduction of biotic homogenization in the introduction (lines 52-57) which gives the reader a better understanding of why we would expect lower turnover and greater representation of generalist species at landfill active sites. Additionally, we have altered the wording in our hypothesis to say “carnivore and scavenger species” rather than omnivorous species, as this more accurately represents our expectations (line 121). We have clarified our reasoning for this expectation using previous literature on landfill bird communities (lines 64-67).

*Particular comments:*

*Line 51: I think that with only say biotic homogenization is enough for this sentence*

We agree, and have removed “of landscapes” from this sentence (line 57).

*Line 85: I would use only citizen science and exclude volunteer-led -science, because the term use thereafter is the abbreviature CS*

“Volunteer-led science” was removed from this sentence (line 104).

*Line 89: it says that the study assessed the patterns of trait diversity of birds, but trait diversity is usually understood as functional diversity and I did not see any metric of functional diversity analysed in the study?. Although diet is a trait, is uncommon in the literature refer to it as trait and ca be confusing. I would suggest changing the term and instead use: dietary guilds, because this was the aspect explored in the study.*

We have changed this sentence to read “taxonomic,habitat, and dietary diversity” (line 108).

*Line 90: it is not necessary to say reported by birdwatchers, because this is already clear in the methodology that the data comes from ebird*

We have removed “reported by birdwatchers” for clarity (line 109)

*Line 91: county-level species pools concept is suddenly introduced in the formulation of the hypothesis, but it could be great to explain in the introduction why is important to compare bird diversity of landfills with county- level species pools.*

We have added a sentence explaining why we compare landfills to county-level species pools (lines 113-116)

*Line 92-93: I consider that in the hypothesis i should be specified what refence sites are the authors referring? because some reference sites (urban) can have similar or even lower levels of taxonomic diversity.*

We added the sentences “Reference sites were sites within the same county as each landfill which had been similarly sampled by birdwatchers, allow for direct comparisons of average species richness and patterns of species turnover, in landfills versus “natural” sites” in the introduction (line 110-113) and sentences addressing the types of reference sites included (lines 165-169), as well as a reference to a supplemental table (S1 Table) which is a list of all reference sites.

*Line 93: It is not clear to me the expression: altered community composition? How can we explicitly define this in highly modified human landscapes? Because in these landscapes almost all bird communities have altered species composition? To make clearer I would more explicitly define in the introduction what are these reference sites.*

We agree that ‘altered community composition’ is vague, and so we have removed this as a standalone hypothesis in the paper, and instead focus on the more specific hypotheses related to species richness and similarity, and diet. We have adjusted the structure of our methods section to reflect this change (line 217-221).

*Lines 93-94: To me higher species similarity and lower turnover are basically referring to the same, it sounds to me redundant. You can leave just one.*

We have removed “and lower turnover across space” from this sentence for clarity (line 119-120).

*Line 95: Again, I think that the part of omnivorous and generalist diets is redundant, because omnivorous are generalist in diet, because they use different food items.*

We have removed “with generalist diets” from this sentence for clarity (line 121).

*Line 96: It is not clear the term “background species pools”. This term is describing county-level species pools, reference sites or both?*

We have changed this sentence to say “county-level species pools” (line 122) and have been consistent in only using this term in the rest of the manuscript, so that it is clear to readers what we are referring to. For clarity, “background species pools” is no longer included in the manuscript.

*Line 98: I consider that besides reference sites it could be added county-level species pools, because in the lines 90 and 91 it says that the study will compare landfill with these two categories.*

This sentence was adjusted to be clear that we compared dietary guilds between landfills and county-level species pools (lines 125-126)

*Materials and methods*

*General comments:*

*1. I consider that the authors should more explicitly give the reasons for not analysing the raw data of the checklists for exploring the taxonomic diversity of the landfill bird assemblages. I think that the argument of use encounter frequency data to avoid biases is weak. First, because in the reference cited is not explained and explicit the way how to analyse bird community data, being more a study aimed to compare vocal activity of one species (see reference 26). Second, because there are several ways to approach these biases of ebird data. For example, to account for observer heterogeneity, authors can calculate observer expertise scores using mixed general additive models (see https://www.nature.com/articles/s41467-020-18230-0) for a recent example. Excluding checklists from observers with low experience can also reduce the bias. Third, use of raw data of the checklists allow for future studies to make comparisons of landfills with the present study. Particularly, for exploring temporal trends in taxonomic diversity. For these reasons, I strongly recommend to the authors to make the analysis of bird data using the original observed species level counts.*

We appreciate the points raised by the reviewer; however, we maintain that the use of the summarized eBird data is appropriate and sufficient for the analyses we wished to conduct; further, the alternative of obtaining, cleaning  and summarizing the raw checklist data suggested here would comprise an entirely new analysis, and calculation of different summary statistics, that is beyond the scope of our original analysis. Since, additionally,  reviewers 1 and 3 took no issue with our approach, we have elected to stick with analysis of the summarized data. Below, we provide further justification of why we chose to work with summarized eBird data and add text to the discussion (lines 391-404) highlighting the pros and cons of using summarized versus raw data.

- We emphasize that summarized eBird data have been used in other studies (e.g. reference 26, which is now reference 31). We have moved this reference to earlier in the sentence (line 146) to reflect this and to clarify that we weren’t suggesting that this study used similar methodology to ours.
- When using citizen science data, there will always be questions about data quality and observer heterogeneity, but the eBird summary data published as encounter frequencies has already been subject to several quality control measures. eBird’s volunteer reviewers verify records of unusual species or atypical counts, and exclude checklists with insufficient documentation or misidentifications from the summary data on eBird’s public site. Within the US, each state has a team of reviewers and in some of the states included in this analysis (e.g. Texas and California) there are multiple reviewers for each county.
- Use of summary data also allows inclusion of species documented only on incomplete checklists, which are often excluded in analyses of raw eBird data. Incomplete checklists are more likely to contain locally rare sightings (e.g. declining habitat specialists and passage migrants), as some birders use eBird only to record unusual sightings rather than all species they encounter(<https://support.ebird.org/en/support/solutions/articles/48000967748-birding-as-your-primary-purpose-and-complete-checklists>). Including, but downweighting occurrence data from incomplete checklists allows for inclusion of these species in species presence analyses without introducing biases in summarized encounter frequencies. We have clarified this point in our revised manuscript (line 142-143). We conducted all of our analyses with both a full list of species recorded at each site (i.e. including data from incomplete checklists), as well as with a more restricted list of species encountered on more than 5% of checklists (lines 196-198), i.e. excluding incomplete checklist data).

*2. While I agree with the use of the Wilcoxon rant test for comparing the differences in species richness between landfills and reference sites because is count data, I wonder to know why the authors did not consider other statistical models to assess the differences between landfills and reference sites. For instance, generalized linear models using a poisson distribution can be used or even GAM models (see   https://rss.onlinelibrary.wiley.com/doi/abs/10.1111/j.1467-9868.2010.00749.x). I consider that the authors should explore the possibility to use other statistical models and not only depended of a single statistical test to support their conclusions or at least provide arguments for not using other statistical models.*

The Wilcoxon rank test is a simple and appropriate statistic for analyzing the difference between these two groups. While other methods are also applicable, the Wilcoxon rank test is simple, familiar and therefore accessible to most  readers. As a result, we have decided to leave this analysis as it is.

*3. Although I agree with the use of the Jaccard index for doing the pairwise comparisons, I think that the other indices can be used as well. For instance, Sorensen index is proved to be useful for exploring community similarity using only incidence data such as Jaccard. However, Sorensen compared the number of shared species to the mean number of species in a single assemblage, giving a more local view than Jaccard which can greatly enrich the exploration of community similarity in the manuscript.*

We repeated this analysis using Sorensen’s Index and found the same result. This analysis is now reported in the supplements (S1 Fig.)

*4. The authors mixed “reference sites” and “county species pools” in the formulation of the hypothesis and their predictions and is not clear whether both are synonyms or different.*

We have adjusted the formulation of the hypotheses to be clearer. We now have three hypotheses. Hypotheses i and ii are comparing landfills to reference sites (lines 118-120) and hypothesis iii compares landfills to county-level species pools (lines 120-122).

*Particular comments:*

*Lines 106-108: Besides these applications, has eBird data been used before to explore the diversity of landfills? if yes would be important to notice this here or perhaps better in the introduction.*

To the best of our knowledge, eBird data has not been used to explore diversity at landfills.

*Lines 109-111: It is not clear how similarly are sampled refence sites in the county to the landfills in terms of sample completeness? It would great if the authors describe in quantitative terms this similarity in sampling effort. For instance, do reference sites of >= 100 checklists were included?*

We have added two sentences describing the similarity of sampling effort (lines 246-249). A paired Wilcoxon test revealed no significant difference between sampling effort at landfills and their corresponding reference sites. All landfills and reference sites in the analyses had over 100 checklists submitted.

*Line 110: This line is a perfect place for defining what does it means county-level species pool. For people in the USA, this is clear but for readers of the rest of the world probably no.*

We have clarified what a county-level species pool is in terms of our analysis (lines 113-116). We have also provided rationale for choosing county level data rather than another scale (lines 177-182). Additionally, we have included data on the area of the counties included in the analysis (lines 251-252), so that readers better understand the scale of the counties we discuss.

*Line 118-119: I sought reference 26 and I checked the material and methods of this publication, but I could not find an explicit and clear explanation about this critical point for the data analysis. I would request the author a better explanation for this data analysis procedure*

Reference 26 (now reference 31) was chosen because it also uses summarized encounter frequency data rather than raw data. We did not choose it because we used similar methods of analysis. We moved this reference earlier in the sentence to clarify this. This is also referenced and highlighted above (Materials and Methods: General Comment #1)

*Lines 125-126: Authors should explain in these lines why is important for sampling bird taxonomic diversity to only have landfills with active sites?. I’m curious about the reason.*

We chose to work only with active landfills for a couple of reasons. First, we were interested to know whether active landfills are used by species other than those scavenging on trash, or whether the presence of trash-feeding species and other human disturbance associated with waste disposal reduces their suitability compared to reference sites. If other habitats at active landfills already host species of conservation concern, then conservation actions can be implemented before the landfill is closed to trash disposal. Second, many birders visit active landfills because the active trash disposal sites can attract unusual species, and so we were able to find many active landfills with a sufficient number of checklists for the analysis. We agree that it would be interesting to see how post-closure landfills differ in their bird communities relative to active landfills and reference sites, and we suggest this as an interesting direction for future work in the discussion (lines 411-416)

*Line 132: There is an additional bracket at LTC034*

The hotspot appears as “South Padre Island (LTC 034)” on eBird, including the bracket. To make this clearer, we have added quotation marks around the hotspot name (lines 164-165).

*Lines 135-136: Here the authors explain better the criteria to select reference sites, but I believe that is a good idea to establish a threshold value of the reference sites to be compared with the landfills.*

We have altered the materials and methods to more clearly state the selection process of the reference sites (lines 159-171). We have explicitly stated the threshold for inclusion (>100 checklists) in line 161.

*Line 138: does county level data exclude the data of the landfills?. I assume that yes, but perhaps this can be detailed more thoughtfully.*

County-level data does not exclude data from landfills, because it is impossible to extract these from the summary data. However, this will not skew the results for the county-level analysis because the landfill data represented a very small portion of the overall county data in eBird; an average of 1.5% (range 0.1-5%) of county checklists were from the landfill in that county.

*Line 142: Mention the year 2014 into the text is redundant because this is clear in the reference*

We have removed “published in 2014” from this sentence (lines 185-186)

*Line 144: It is a good idea to separate the category “FruiNect.”, because it would be important to explore how frugivorous and nectarivores guilds are independently responding to lanfdills. Merge them in a single category can hide these responses. This separation is possible in the Whitman et al 2014 database*

Given the fact that the combined *FruiNect* category represented no more than 1.5% of overall observations (figures 4 and S2), we have decided to not separate these two categories.

*Lines 152-153: I recommend justifying with a reference the threshold value of 5% for the encounter frequency*

We do not have a reference to justify our choice of threshold; however, we explored the sensitivity of our results to the cutoff threshold value. We tested 1%, 2.5%, 5%, and 10% and have presented these results here. First, we present a table with the results of Wilcoxon Rank Tests on the difference in species richness between landfills and reference sites. lfSR represents the median species richness for landfills and reSR represents the same for reference sites. These results show a small difference in medians between groups for every threshold except 5%.

Next, we present the community similarity over distance graphs for each threshold value. The values for Jaccard’s Index decreases as the threshold for commonness increases, but we see no change in difference between groups at any threshold value.

Finally, we believe that the threshold value of 5% is most suitable to exclude chance sightings (e.g. flyovers), to represent only the “most commonly encountered species.” Given that our threshold for including a site was a minimum of 100 checklists, a threshold of 1% would mean a species that was seen only one time would erroneously be included in the “most common species.” Our analysis above suggests that a higher threshold of 10% is too restrictive, reducing median species richness at landfills from 145 (all species) to 42 (10%).

*Lines 167-171: It is not clear how site-specific species-level encounter frequencies are used in the non-metric multidimensional scaling (NMDS) analysis to explore the degree of overlapping between clusters. I agree with the use of NMDS and ANOSIN to tests this prediction, but I think that this point (how frequencies are used and visualized in the NMDS) should be explicitly described.*

Our understanding is that, as a non-parametric rank-order method, NMDS is suitable for numerous data types, including the encounter frequencies used here. We have clarified that encounter frequencies were used to calculate a dissimilarity metric for each landfill-reference site pair within a given county (lines 215-216).

*Lines 182-184: why should we expect a shallower relationship for landfills?. I recommend the authors explain this in the introduction to give context about these relationships.*

We have edited the introduction to better explain the concept of biotic homogenization (lines 52-57) why we would expect a shallower relationship for landfills (lines 64-67).

*Lines 186-189: why landfills are only compared with county level species pools?*

Landfills are compared to county-level pools, due to landfills representing one habitat type, meanwhile reference sites across the country can be different habitat types (i.e. lakes, forested natural sites, urban parks, etc.)

***Results:***

***General comments:***

*To me is not clear the results presented in the lines 241-251. Are these representative counties reference sites? or county level species pools? In the text is ambiguous. Figure says county level species pools. Authors should clarify this point. Furthermore, it is not clear why dietary guild comparison is not made with reference sites, if the core of the study is the comparison between landfills and reference sites.*

We have edited the text to be clearer that we are comparing landfills to county-level species pools (line 300). We have added various sentences (lines 113-116;177-182) to clarify the reasoning for comparing landfills and county-level species pools as is discussed in other sections of this document.

***Particular comments:***

*Line 194: Only say dataset is enough. It would omit “data” in this part. Otherwise sounds redundant.*

We have made this adjustment (line 244)

*Lines 212-214: It is not clear why there is pseudoreplication in the pairwise comparisons? In the lines 197 and 198, the authors say that the mean distance between pair landfills and reference sites was 18.2 km. Based in this mean I think that at least some points are enough spatially isolated from each other to avoid pseudoreplication or what is the nature of the pseudoreplication in this comparison. I’m lost in this part; I would like to hear an explanation of the authors.*

The problem is not a lack of independence of the points, but the fact that each data point is used many times: each site must be compared to all other sites, inflating the N. Based on this comment, we have omitted the term "psuedoreplication" (line 267) from the text because it may be misleading.

*Lines 214-215: Why there was no evidence that species similarity between landfill pairs was higher or decayed more slowly with inter-site distance than between reference site pairs? Which part of the results support this? Because there was a not formal statical test of this aspect.*

We have altered our text to reflect that no formal statistical test was performed to draw our conclusion (lines 268-269), but that inspection of the plotted relationships suggests no differences in the decay of species similarity with distance between the two site types. We also repeated the analysis using Sorensen’s Index and include these results in the supplements (S1 Fig)

*Line 226: The next sentence: “geom_smooth 226 function and ‘loess’ method in ggplot” is unnecessary to include in the explanation of the figure 3.*

These terms were removed from the sentence (lines 282-283)

*Line 227: I consider reassess this title, because species richness is one facet (measure) of taxonomic diversity) and this already covered in the previous section and trait diversity see previous comment. In fact, this part looks more concerned to species composition.*

We have changed the title of this section to “indicator species analysis and dietary diversity” (line 284-285).

*Line 236: It could be a good idea in the table 1, specify the category column, is referring to the degree of habitat or diet specialization? Or both? Perhaps the column “reference” should be called “reference sites”?*

We have corrected the title of the “group” column to be now “habitat specialization” and changed the “reference” column to “reference site” (Table 1).

*Line 242: what are the representative counties? Reference sites or county level species pool?*

We have edited this sentence to say “county-level species pool” so that it is clear what we have compared (line 300).

*Lines 248: Correct “granviores”*

We have corrected the spelling (line 306).

***Discussion:***

***General comments***

*1.*      *Is missing in the discussion the factors (ecological, human) that can drive the not divergence in species richness between landfills and reference sites. I suggest the author focus more in discuss these factors and much less in previous studies in landfills not directly related to the aim of the study.*

We have removed several sentences in accordance with the particular comments and included more discussion of the ecological and human drivers of landfill bird communities.

*2.* *Author should consider discussing more why some particular groups as gulls are benefitting of landfills compared to other urban human land uses? This group showed a positive response to landfills but is not discussed.*

We have added a sentence clarifying that gulls and starlings also benefit from other human activities, including other urban land uses (line 337-340). We mention that being both habitat and dietary generalists, they thrive in human adapted environments, including landfills.

*3. Foraging guilds such as nectarivores and frugivorous are not discussed, do these groups showed a particular prevalence to landfills? If not, why.*

We found a very small (<1%) absolute difference between frequencies of frugivorous and nectarivorous guilds at landfills and their expected frequency at county-level species pools, with these guilds being scarce compared to other diet types (<1.35%). As a result, we have not included discussion of this point.

*4. Some taxonomic groups are missed in the discussion. Besides gulls, waterfowl and some grassland species, particular passerine families (Passeriformes are the most abundant and diverse bird order) showed differences between landfills and reference sites?*

Because of difficulties in calculating and interpreting species-level encounter frequency data at higher taxonomic resolution, we could not conduct any family-level or order-level analyses and only report the species whose frequencies differed at landfills in the indicator species analyses. We mention in the discussion (lines 396-399) that these broader scale taxonomic analyses would require analysis of abundance data.

*5. How this study benefits from comparing landfills with county species pools? County species pools are missed in the discussion.*

We have edited the first paragraph of the discussion to more clearly explain what our county species pool analysis showed (lines 329-333). This analysis showed that scavengers and carnivores are encountered more frequently at landfills than would be expected, given their background encounter frequency.

*6.*      *I missed a general conclusion and take-home message for this study. The author should clearly develop a conclusion at the end of the discussion and not only highlight knowledge gaps and future research work.*

We have developed a conclusion with a strong and clear take-home message for the study (lines 422-428)

***Particular comments:***

*Line 260: To me was not evident the comparison of landfills with county-level species pools. I consider that that the discussion mainly be focused to the comparison between landfills and reference sites because this what is observed in the results.*

We have addressed these concerns in other parts of this document and in the introduction and methods of the revised manuscript. Additionally, we added a sentence to the discussion explaining our findings of the county-level analysis (lines 329-331).

*Line 261-262: I think that this point is dubious, because either landfills and reference sites are inside the urban matrix, and it not clear why resource subsidies reduce species richness.?*

We are suggesting that the resource subsidies available at landfills (human garbage, rotting food, rodents, etc.) will attract a similar suite of species like gulls, vultures, and other scavengers. In accordance with this comment and others, we have more clearly explained our rationale for our hypotheses in the introduction (lines 52-54; 61-70). Additionally, we clarified this point in the discussion (lines 320-322), adding “landfills would be less speciose, because they are dominated by dietary generalists and widespread human-adapted species that outcompete habitat specialists.”

*Line 263: Again, I would suggest using a strong statistical model to test this comparison.*

We have addressed this point earlier in this document (Materials and Methods: General Comment #2).

*Line 264-265: The phrasing of is part is not clear. I would rephrase it again and point out that species turnover was not pronounced in landfills compared to pairwise comparisons of refence sites. Because this is what is shown in the figure C.*

We have clarified the phrasing so that it states we found no differences between pairwise comparisons of landfills and pairwise comparisons of reference sites (lines 324-325)

*Line 268-270: I think that the authors should be more cautious with this conclusion. First, because of the quality and nature of the ebird data, second, because of the temporal scale of the checklists surveyed. Third, because a null model expectation was not rule out, it not possible to ascertain whether these differences actually exists. Fourth, because of the geographical bias, many locations strongly concentrated in the north-eastern region of the country, the small difference detected by the statistical tests etc. I would leave as a possibility, “probably”.*

We agree with the many caveats of this conclusion, so we have added the word “may” to this sentence (line 331-334). The sentence now reads “landfills may have the potential,” which more clearly states it is a possibility, not a definitive statement that landfills have the same diversity as natural communities.

*Lines 273-277: from line 273 to 277 the information presented is not relevant for the discussion of the results of this study, I did not see any connection bird-aircraft collisions and increased disease risk with the aim of this study (compare taxonomic bird diversity of landfills and reference sites). I suggest omit this part and focus more in discuss a little bit more why gulls and starling are thriving in landfills?*

We have removed this information from our revised manuscript. We have instead included discussion of why gulls and starlings thrive at landfills (lines 337-340).

*Line 275.277: If there is not enough previous references to discuss the results , is better to avoid statements about the not utility of the literature and instead highlight knowledge gaps that can be explored by future research to understand better the value of landfills for conserving birds.*

We have removed this sentence and highlight knowledge gaps later in the discussion (lines 413-421)

*Lines 280-282: How the results of this study are consistent a positive correlation between habitat diversity and bird species richness in human-modified urban parks, when habitat diversity was not measure as the author claimed? Because habitat was not measured in this study, it hard to support that this study is proving evidence for the correlation between habitat heterogeneity and bird diversity*

We have edited this sentence to clarify (lines 349-352). We state that we did not test habitat diversity, but that our findings are consistent with the hypothesis that the variety of habitat types found at landfills may be responsible for diverse bird communities. Then we say that similar relationships have been found in human-modified environments (urban parks) before.

*Line 282: Avoid starting a phrase with “of “*

We have reworded this sentence to start with “Interestingly” (line 352)

*Lines 287-288: I think that this part: “These areas are regularly mowed to prevent the growth of deep-rooting species, which are believed to be able to pierce the containment system “is irrelevant for the present study*

We have deleted this sentence.

*Line 289: Try to include also scientific names for the species mentioned in the text. Otherwise common names will not prove to be helpful when researchers search for information of particular species in searches using web of science, scopus or google scholar or directly into the papers and reviews etc.*

We have added the scientific names for all species mentioned in the text and in Table 1.

*Line 299: I ask: this part refer to suitable population declines of grassland birds? In that is the case, authors can make it more explicit*

        	This sentence is referring to declines in grassland bird habitat, not in grassland bird populations.

*Line 307: I think that one of the main assets of this study is highlighted here. This study makes a continental scale. This should be more clearly highlighted in the introduction section.*

We have added the word “continental” to the introduction to clarify that our study used sites spanning the continental United States (line 109).

*Lines 318-319: See comments in the methodology about this point.*

We have addressed these concerns in other parts of this document.

*Line 321: I would not only say eBird but “citizen science initiatives” such as inaturalist, xenocanto etc.*

We made this adjustment (line 388).

*Line 326-327: in the lines 301-303 this was already discussed, sounds a little bit repetitive*

We have removed the sentences that were repetitive

***References***

*1. Avoid using hyperlinks to the cited references, see references: 7,44*

*2. Avoid capitalize the titles and names of the journals, see references: 14, 23,34 ,44*

*3. Avoid underlie, see references: 7,14,18, 30,43,44*

 	We have addressed all of the style issues with the references that are addressed here. Additionally, we made sure to maintain the same style with all new references.

***Figures***

*Avoid white lines in the background of the figures*

We have removed the white lines in the background of the figures.

*Include the description for the dietary categories used in the figure 4. For example, explain what it means* *VertFishScav* *etc.*

We have included an extra sentence in the methods defining VertFishScav, as defined by the EltonTraits database (line 188).

**Reviewer #3:**

*This manuscript addresses an interesting question about bird community on landfills, these waste disposal areas normally are focus of studies investigating diseases risks with its associated bird species considered generalists and homogeneous than surroundings. The authors used a citizen science based data from eBird to compare richness and community similarity between landfills and reference sites. The methods were clear and well developed to standardize samples by checklists, stating limitations and biases related to site selection and clearly taking account during interpretation of results and conclusions. All conclusions were based on data analyzed with implications to direct management of landfills to improve its role in maintain habitat specific and threatened species. In general, the manuscript is well written, all data are publicly available, provide new insights about bird community of a few studied anthropogenic landscape, and an interesting title with high potential to be used on scientific communication texts. Based on characteristics stated above I recommend publish as it is.*

We are grateful to the third reviewer for this positive review.
